# Family screening for abdominal aortic aneurysms: a systematic review and meta-analysis

**DOI:** 10.1016/j.eclinm.2026.104056

**Published:** 2026-07-09

**Authors:** Leonardo Buttice, Jonathan J.H. Bray, Claire E. Foster-Archibald, Sophie E. Thompson, Dominic Howard, Alex Pitcher

**Affiliations:** a Torbay Hospital, Newton Road, Torquay, Devon, TQ2 7AA, UK; b University College London, Gower Street, London, WC1E 6AE, UK; c Buckinghamshire Health Research and Innovation Centre, Stoke Mandeville Hospital, Aylesbury, HP21 8AL, UK; d Hereford County Hospital, Stonebow Road, Hereford, HR1 2ER, UK; e Department of Cardiovascular Sciences, School of Medical Sciences, College of Medicine and Health, University of Birmingham, Birmingham, B15 2TT, UK; f Wolfson Centre for Prevention of Stroke and Dementia, Nuffield Dept. of Clinical Neurosciences, University of Oxford, Oxford, OX3 9DU, UK; g Department of Vascular Surgery, Oxford University Hospitals NHS Trust, Oxford, OX3 9DU, UK; h Oxford Heart Centre, John Radcliffe Hospital, Headley Way, Headington, Oxford, OX3 9DU, UK

**Keywords:** Abdominal aortic aneurysm, Family screening, Prevalence, First-degree relatives, Familial

## Abstract

**Background:**

International guidelines recommend screening of first-degree relatives of patients with abdominal aortic aneurysm (AAA). However, the prevalence of AAA amongst screened relatives is uncertain.

**Methods:**

Medline, Embase, and the Cochrane Library were searched from inception to 4 March 2026 for studies screening relatives of patients with AAA. Data were analysed using a random-effects model meta-analysis in Stata. Risk of bias was assessed using a validated tool for prevalence studies. Evidence quality was assessed using GRADE. Aims and analyses to be performed were pre-specified and recorded with PROSPERO: CRD42024566370.

**Findings:**

Twenty-six studies were included with 4166 AAA index patients and 4530 screened relatives, providing moderate-certainty evidence. The prevalence of AAA amongst relatives overall was 14.4% (95% CI 10.7%–18.4%) (24 studies), 21.9% (95% CI 15.9%–28.6%) in male relatives (19 studies), and 6.3% (95% CI 3.6%–9.5%) in female relatives (17 studies). The prevalence of familial AAA, where AAA index patients had at least one affected relative, was 16.9% (95% CI 12.7%–21.6%) (13 studies). The odds of finding an AAA amongst male relatives was about 4 times greater than in female relatives (OR 3.69, 95% CI 2.89–4.71). Screened female relatives had a 5 times greater risk of having AAA compared to age- and sex-matched controls, whereas the comparable figure for male relatives was about 3. The risk of bias was low for all studies.

**Interpretation:**

Relatives of patients with AAA are a highly enriched population for AAA, occurring in 1 in 5 males and 1 in 16 females, with 1 in 6 AAA index patients having at least one affected relative. These findings support screening strategies that encompass all first-degree relatives, both male and female, irrespective of reported family history in the AAA index patient.

**Funding:**

None.

**Research in context**

**Evidence before this study**

International guidelines recommend screening of first-degree relatives of patients with abdominal aortic aneurysm (AAA). However, this is not routinely performed in current screening programmes. This is in part due to uncertainty about the prevalence of AAA in family members. This systematic review and meta-analysis aims to collate and evaluate reported prevalence data to provide a more precise and reliable prevalence of AAA in relatives of patients with AAA.

**Added value of this study**

Medline, Embase and the Cochrane library were searched from inception to 4 March 2026 for all studies where relatives of patients with AAA were screened. Case reports and studies exclusively investigating rare syndromic disorders were excluded due to their enrichment for AAA. Twenty-six studies, including 4166 AAA index patients and 4530 screened relatives, were identified. Meta-analysis found that screened relatives are a substantially enriched population with 1 in 5 screened males and 1 in 16 screened females being found to have AAA, and about 1 in 6 AAA index patients screened had familial AAA, with at least one affected relative. Additionally, female relatives have a particularly high prevalence, compared to females with no family history (5 times higher), despite not currently being eligible for systematic screening in some national screening programmes.

**Implications of all the available evidence**

Our findings demonstrate that relatives screened from the age of early 50s to late 60s are a highly enriched population for the detection of AAA. Systematic screening of all first-degree relatives (of either sex), irrespective of reported family history in the AAA index patient, is likely to be a productive strategy for the early identification of individuals with AAA.

## Introduction

Abdominal aortic aneurysm (AAA) is a potentially life-threatening condition responsible for over 4000 deaths in England and Wales in 2019, accounting for 1.7% of all deaths in men aged 65 years or older.^1^ Due to the prevalence, high rupture associated mortality rate^2^^,^^3^ and the need to intervene early,^4^ several countries adopted national screening programmes, with the United Kingdom (UK), Sweden and Germany offering a single ultrasound scan to all men aged 65 or over and the United States of America (USA) offering it to men aged 65–75 who have ever smoked.^5^, ^6^, ^7^, ^8^ Ontario, Canada has also begun a provincial screening programme, scanning men and women aged 65 or older.^9^ However, it is apparent that the prevalence has been declining, bringing into question the usefulness and cost-effectiveness of population screening.^10^^,^^11^

Familial aggregation of AAA has been consistently demonstrated in family history^12^, ^13^, ^14^, ^15^ and screening studies,^16^, ^17^, ^18^, ^19^, ^20^, ^21^ supporting genetic susceptibility as a major risk factor for AAA development. Approximately one in five patients with AAA report an affected relative,^12^, ^13^, ^14^, ^15^ and screening studies have shown increased prevalence among first-degree relatives compared with the general population.^16^, ^17^, ^18^, ^19^, ^20^, ^21^ Already in 1977, Clifton observed familial clustering of AAA in three brothers, suggesting a familial predisposition to the condition.^22^ Twin studies and genetic analyses have since provided further evidence for the heritability of AAA,^23^^,^^24^ with aneurysm-associated genetic variants estimated to account for approximately 5% of cases,^15^^,^^25^ and new susceptibility genes continue to be identified.^26^

To address the declining prevalence, targeted screening of high-risk populations has been proposed, with several international guidelines now recommending screening of first-degree relatives (FDRs), regardless of sex.^27^, ^28^, ^29^, ^30^ However, this is not consistently adopted in routine practice.^31^ Moreover, given cut-offs for AAA used today were originally derived from male populations, more work could be done to understand female-specific cut-offs.

This meta-analysis aimed to provide a more precise estimate of AAA prevalence in screened relatives, in order to improve prediction of familial genetic risk, inform screening recommendations and provide sex-specific prevalence estimates for screening counselling.

## Methods

This systematic review and meta-analysis was written in accordance with the preferred reporting items for systematic reviews and meta-analyses (PRISMA) 2020 guidelines^32^ (Supplementary Table S1) and registered with PROSPERO (CRD42024566370),^33^ pre-specifying the aims and analyses to be performed. Ethical approval was not required as no patient-identifiable data was used.

### Search strategy

The PICO framework was used to structure the research question (Supplementary Table S2). A search of the Cochrane Library, Embase and Medline from inception to 4 March 2026 was performed searching for studies where relatives of patients with AAA were screened to identify new AAA cases. Where feasible, medical subject heading (MeSH) terms were used. Duplicates were removed using EndNote 20, and two reviewers (LB and SET) independently screened the search results before commencing full-text eligibility assessments. The references of eligible studies were checked for missed studies. Inconsistencies were resolved by consensus using a third reviewer (JJHB) reviewing the full text (Supplementary Table S3). Google Translate, and where possible, colleague spot check, was used to translate included studies into English.

### Eligibility criteria

Eligible studies included those that identified a group of patients with AAA and then invited their relatives for AAA screening. No limits were set regarding study type (observational, randomised-controlled, case series), country, language, screening modality, type of patient with AAA (sporadic versus familial), type of relative (first-degree, siblings, offspring, second-degree), age, sex or timing of screening. Case reports and studies specifically investigating AAA in rare syndromic disorders, e.g. Marfan syndrome and Ehlers-Danlos, were excluded, as these populations are enriched for AAA.^34^^,^^35^ Thoracic and peripheral aneurysm data were excluded, as they fall outside the scope of this review. Studies not using imaging to screen relatives were excluded. Studies were also excluded if they did not include data that could be quantitatively analysed using meta-analysis.

### Data extraction

Data from eligible studies were independently extracted into a pre-formatted Excel spreadsheet (Microsoft Office 365) (LB) and checked by a second reviewer (JJHB). Data included study design (timing, country/centre, screening modality), participant characteristics (type of relative, numbers, age, sex) and the prevalence of AAA amongst those screened (Table 1).

**Table 1:** Table of study designs and baseline characteristics.

First author	Year	Country	Method of invitation	Relative type	Screening method	Relatives invited/relatives attended (%)	Total participant numbers (patients/relatives)	Age of patients/relatives (years)	Sex of patients/relatives (% male)
J. Collin^16^	1989	UK	–	Siblings	USS	87/32 (36.8)	140 (108/32)	−/−	94.5/53.1
H. Bengtsson^36^	1989	Sweden	–	Siblings	USS	102/87 (85.3)	168 (81/87)	−/61.8	78.6/40.2
C. Pecis^37^	1991	Italy	–	FDRs	USS	–	93 (44/49)	−/−	−/−
M. W. Webster (USS)^38^	1991	USA	Telephone	FDRs	USS	202/110 (54.0)	153 (43/110)	−/−	−/−
J. Adamson^39^	1992	UK	–	Siblings	USS	57/53 (93.0)	81 (28/53)	−/64.0	89.3/47.2
H. Bengtsson^40^	1992	Sweden	–	Offspring	USS	90/62 (68.9)	217 (155/62)	−/60.1	−/62.9
D. Moher^41^	1992	Canada	–	Siblings	USS	–	>48 (−/48)	−/65.2	−/100
A. van der Lugt^42^	1992	Netherlands	Letter/telephone	Siblings	USS	128/108 (84.4)	140 (32/108)	62.0/60.0	87.5/51.9
D. C. Adams^17^	1993	UK	Letter	FDRs	USS	110/76 (69.1)	168 (92/76)	−/−	−/50.0
F. Speziale^43^	1994	Italy	–	FDRs	USS	173/101 (58.4)	152 (51/101)	−/−	−/−
P. A. Baird^44^	1995	Canada	–	Siblings	USS	54/54 (100)	180 (126/54)	−/−	83.3/48.2
P. Fitzgerald^45^	1995	Ireland	Letter	Siblings	USS	234/125 (53.4)	245 (120/125)	71.5/62.1	72.5/48.0
G. Larcos^46^	1995	Australia	Letter	FDRs	USS	–	182 (130/52)	−/54.0	−/50.0
P. Jaakkola^47^	1996	Finland	Letter	Siblings	USS	–	279 (148/131)	−/−	−/38.9
Y. van der Graaf^48^	1998	Netherlands	Telephone	Brothers	USS	269/223 (82.9)	514 (291/223)	69.0/−	89.7/100
J. A. Salo^18^	1999	Finland	–	FDRs	USS	325/241 (74.2)	391 (150/241)	70.4/65.2	78.6/41.9
C. J. van Keulen^49^	2000	Netherlands	–	FDRs	USS	176/82 (46.6)	110 (28/82)	69.0/53.2	−/61.0
J. I. Rossaak^50^	2001	New Zealand	–	FDRs	USS	–	82 (33/49)	−/55.8	−/−
G. Frydman^19^	2003	Australia	–	Siblings	USS	406/276 (68.0)	676 (400/276)	69.8/66.0	79.0/54.0
T. Ogata^20^	2005	Canada	Telephone	Siblings	USS	606/333 (55.0)	708 (375/333)	72.1/69.4	77.7/45.7
S. A. Badger^51^	2007	Ireland	Letter	FDRs	USS	405/320 (79.0)	432 (132/300)	−/63.3	68.0/52.3
A. Linne^52^	2012	Sweden	Letter	Siblings	USS	216/150 (69.4)	562 (412/150)	−/66.3	−/44.0
N. Sakalihasan^53^	2014	Belgium	Letter	FDRs + SDRs	USS	510/186 (36.5)	508 (322/186)	69.0/63.0	92.0/45.9
A. Linne^21^	2016	Sweden	–	Siblings	USS	663/529 (79.8)	1279 (750/529)	−/67.0	−/48.0
N. Fattahi^54^	2024	Sweden	–	Offspring	USS	1126/752 (66.8)	>752 (−/752)	−/64.1	−/46.5
H. Liu^55^	2024	Netherlands	–	FDRs + SDRs	Non-contrast CT	–	416 (115/301)	69.0/57.8	77.4/47.5

“–“ denotes data not available.

CT, computed tomography; FDRs, first-degree relatives; SDRs, second-degree relatives; USA, United States of America; USS, ultrasound scan; UK, United Kingdom.

Data were collected based on the prevalence of AAA amongst all screened relatives, and where possible, reported male-to-female ratios were used to compare the prevalence of AAA amongst male-only relatives and female-only relatives. Data were also collected on the prevalence of familial AAA, where AAA index patients had at least one affected relative. AAA data were collected, provided the measured dilatation was greater than or equal to 2.5 cm. Although AAA is diagnosed at 3 cm, an ectatic/sub-aneurysmal aorta of 2.5–2.9 cm is abnormal, and the Society for Vascular Surgery suggests ongoing surveillance screening due to the risk of developing into AAA, particularly in smokers and patients with COPD.^56^ Comparisons between different measurement cutoffs were done in subgroup analysis (Table 2). Overall prevalences pooled all types of screened relatives.

**Table 2:** Table showing the subgroup and sensitivity analyses performed on the four key outcomes: individual prevalence of abdominal aortic aneurysms in screened family members (All relatives AAA prevalence), prevalence of abdominal aortic aneurysms in male screened family members (Male relatives AAA prevalence), prevalence of abdominal aortic aneurysms in female screened family members (Female relatives AAA prevalence) and prevalence of AAA index patients with at least one affected relative (Familial AAA prevalence).

Primary outcome	Variable	Prevalence % (95% CI)	Subgroup I^2^%
Subgroup analysis			
All relatives AAA prevalence	Includes AAA ≥2.5 cm, n = 8^17^^,^^21^^,^^39^^,^^43^^,^^46^^,^^52^^,^^54^^,^^55^	15.8 (7.9–25.9)	96.21
	Includes AAA ≥3.0 cm, n = 11^19^^,^^20^^,^^36^^,^^38^^,^^40^^,^^42^^,^^45^^,^^49^, ^50^, ^51^^,^^53^	14.1 (10.6–18.0)	77.37
Male relatives AAA prevalence	Includes AAA ≥2.5 cm, n = 7^17^^,^^21^^,^^39^^,^^46^^,^^52^^,^^54^^,^^55^	21.8 (9.8–36.7)	95.02
	Includes AAA ≥3.0 cm, n = 8^20^^,^^36^^,^^40^, ^41^, ^42^^,^^45^^,^^48^^,^^49^	21.1 (16.1–26.6)	57.14
Female relatives AAA prevalence	Includes AAA ≥2.5 cm, n = 7^17^^,^^21^^,^^39^^,^^46^^,^^52^^,^^54^^,^^55^	7.6 (2.6–14.4)	89.26
	Includes AAA ≥3.0 cm, n = 6^20^^,^^36^^,^^40^^,^^42^^,^^45^^,^^49^	6.5 (3.9–9.6)	10.38
Familial AAA prevalence	Includes AAA ≥2.5 cm, n = 2^17^^,^^43^	19.2 (13.1–26.2)	–
	Includes AAA ≥3.0 cm, n = 8^19^^,^^36^^,^^38^^,^^40^^,^^42^^,^^45^^,^^49^^,^^51^	18.5 (12.9–24.7)	71.42
All relatives AAA prevalence	FDRs, n = 9^17^^,^^18^^,^^37^^,^^38^^,^^43^^,^^46^^,^^49^, ^50^, ^51^	12.8 (9.1–17.0)	69.36
	Siblings, n = 11^16^^,^^19^, ^20^, ^21^^,^^36^^,^^39^^,^^42^^,^^44^^,^^45^^,^^47^^,^^52^	16.0 (12.1–20.3)	80.02
	Offspring, n = 2^40^^,^^54^	2.3 (1.3–3.5)	–
	FDRs + SDRs, n = 2^53^^,^^55^	22.3 (18.7–26.1)	–
Male relatives AAA prevalence	FDRs, n = 4^17^^,^^18^^,^^46^^,^^49^	19.8 (10.9–30.4)	64.86
	Siblings, n = 12^16^^,^^20^^,^^21^^,^^36^^,^^39^^,^^41^^,^^42^^,^^44^^,^^45^^,^^47^^,^^48^^,^^52^	22.9 (18.2–28.0)	63.77
	Offspring, n = 2^40^^,^^54^	4.1 (2.2–6.5)	–
	FDRs + SDRs, n = 1^55^	–	–
Female relatives AAA prevalence	FDRs, n = 4^17^^,^^18^^,^^46^^,^^49^	8.9 (3.6–16.0)	51.06
	Siblings, n = 10^16^^,^^20^^,^^21^^,^^36^^,^^39^^,^^42^^,^^44^^,^^45^^,^^47^^,^^52^	6.1 (4.1–8.3)	20.30
	Offspring, n = 2^40^^,^^54^	0.2 (0.0–1.3)	–
	FDRs + SDRs, n = 1^55^	–	–
Familial AAA prevalence	FDRs, n = 7^17^^,^^18^^,^^37^^,^^38^^,^^43^^,^^49^^,^^51^	15.3 (9.3–22.4)	73.67
	Siblings, n = 5^19^^,^^36^^,^^42^^,^^45^^,^^47^	19.9 (12.2–28.8)	78.61
	Offspring, n = 1^40^	–	–
	FDRs + SDRs, n = 0	–	–
Sensitivity analysis			
All relatives AAA prevalence	Excludes studies with AAA index patients with previously known familial AAA, n = 15	13.5 (8.9–18.8)	90.35
Male relatives AAA prevalence	Excludes studies with AAA index patients with previously known familial AAA, n = 14	22.0 (14.7–30.4)	88.16
Female relatives AAA prevalence	Excludes studies with AAA index patients with previously known familial AAA, n = 13	5.8 (2.8–9.5)	76.03
Familial AAA prevalence	Excludes studies with AAA index patients with previously known familial AAA, n = 7	16.2 (9.9–23.7)	74.10
All relatives AAA prevalence	Fixed effect analysis, n = 24^16^, ^17^, ^18^, ^19^, ^20^, ^21^^,^^36^, ^37^, ^38^, ^39^, ^40^^,^^42^, ^43^, ^44^, ^45^, ^46^, ^47^^,^^49^, ^50^, ^51^, ^52^, ^53^, ^54^, ^55^	12.0 (11.0–13.0)	N/a
Male relatives AAA prevalence	Fixed effect analysis, n = 19^16^, ^17^, ^18^^,^^20^^,^^21^^,^^36^^,^^39^, ^40^, ^41^, ^42^^,^^44^, ^45^, ^46^, ^47^, ^48^, ^49^^,^^52^^,^^54^^,^^55^	17.1 (15.3–19.0)	N/a
Female relatives AAA prevalence	Fixed effect analysis, n = 17^16^, ^17^, ^18^^,^^20^^,^^21^^,^^36^^,^^39^^,^^40^^,^^42^^,^^44^, ^45^, ^46^, ^47^^,^^49^^,^^52^^,^^54^^,^^55^	4.9 (3.8–6.1)	N/a
Familial AAA prevalence	Fixed effect analysis, n = 13^17^, ^18^, ^19^^,^^36^, ^37^, ^38^^,^^40^^,^^42^^,^^43^^,^^45^^,^^47^^,^^49^^,^^51^	14.6 (12.6–16.7)	N/a
All relatives AAA prevalence	Excludes overlapping studies, n = 23^16^, ^17^, ^18^, ^19^, ^20^, ^21^^,^^36^, ^37^, ^38^, ^39^, ^40^^,^^42^, ^43^, ^44^, ^45^, ^46^, ^47^^,^^49^, ^50^, ^51^^,^^53^, ^54^, ^55^	14.6 (10.8–18.8)	91.82
Male relatives AAA prevalence	Excludes overlapping studies, n = 17^16^, ^17^, ^18^^,^^20^^,^^21^^,^^36^^,^^39^, ^40^, ^41^, ^42^^,^^45^, ^46^, ^47^, ^48^, ^49^^,^^54^^,^^55^	22.1 (15.6–29.3)	90.03
Female relatives AAA prevalence	Excludes overlapping studies, n = 16^16^, ^17^, ^18^^,^^20^^,^^21^^,^^36^^,^^39^^,^^40^^,^^42^^,^^44^, ^45^, ^46^, ^47^^,^^49^^,^^54^^,^^55^	6.3 (3.5–9.8)	79.37
Familial AAA prevalence	No overlapping studies	–	–
All relatives AAA prevalence	Non-imaging studies, n = 6^13^, ^14^, ^15^^,^^57^, ^58^, ^59^	7.0 (2.4–13.7)	98.59
Male relatives AAA prevalence	Non-imaging studies, n = 2^13^^,^^59^	25.6 (23.2–28.1)	–
Female relatives AAA prevalence	Non-imaging studies, n = 2^13^^,^^59^	2.6 (1.7–3.8)	–
Familial AAA prevalence	Non-imaging studies, n = 9^12^, ^13^, ^14^, ^15^^,^^57^, ^58^, ^59^, ^60^, ^61^	19.6 (15.7–23.9)	81.31

“n” denotes the number of studies. “–” denotes analysis not possible. AAA, abdominal aortic aneurysm; CI, confidence interval; FDRs, first-degree relatives; SDRs, second-degree relatives.

Where control data were reported, data were collected relating to the prevalence of AAA amongst screened controls, as well as the prevalence amongst male-only controls and female-only controls. This would allow comparison with established population prevalence data to help assess the generalisability of the included cohorts.

### Outcomes of interest

The primary outcome was the pooled prevalence of AAA in all screened relatives of patients with AAA. The secondary outcomes were the pooled prevalences in male-only and female-only relatives, as well as the prevalence of familial AAA, where an index patient had at least one affected relative.

Subgroup analyses were performed stratifying studies by AAA measurement cutoffs (≥2.5 cm versus ≥3 cm), and the type of relative: first-degree, first and second-degree, siblings and offspring. Sensitivity analyses were performed, examining the outcomes by removing enriched cohorts such as those including AAA index patients who had relatives previously known to have AAA (familial AAA), by using fixed-effects analysis, by removing studies which overlap populations, by risk of bias, and by assessing prevalence against time and subgroups of country of study origin. Additionally, control data were collected relating to the prevalence of AAA amongst all screened controls, as well as male-to-female ratios to allow for comparison to prevalence rates reported in the literature.

### Statistical analysis

Pooled prevalence and heterogeneity were calculated using the *metaprop* command, a random-effects model meta-analysis in Stata (19.5, StataCorp LLC, College Station, TX, USA).^62^ Freeman-Tukey Double Arcsine Transformation was used to stabilise variances.^63^ The I^2^ measure and p value from the chi-squared test were used to quantify heterogeneity, where I^2^ greater than 50% may represent moderate-to-high heterogeneity.^64^ As per our protocol, we chose *a priori* to use random effects meta-analysis due to the likelihood of clinical and methodological diversity.^33^ In outcomes that included more than 10 studies, funnel plots and Egger's test were used to assess for small study bias.^65^ The *meta esize* command was used to estimate the odds (odds ratio [OR]) of finding AAA between sexes. Descriptive statistics are reported as weighted means ± standard deviation. To explore potential sources of heterogeneity, meta-regression was performed using the *meta regress* command, utilising the default restricted maximum likelihood method. Study-level covariates were included (screened age, sex, smoker, diabetes or hypertension) where there were sufficient numbers of reporting studies (≥10).

### GRADE

A validated risk of bias assessment tool, used to assess prevalence studies, created by Hoy et al.,^66^ was used, where an overall score of 7–10, 4–6 and 0–3 reflects a ‘low’, ‘moderate’ and ‘high’ risk of bias, respectively (Supplementary Table S4). GRADE was assessed using a modified GRADE approach for prevalence meta-analyses, assessing risk of bias, inconsistency, indirectness, imprecision and other bias such as publication bias.^67^

### Role of the funding source

There was no funding source for this study.

## Results

**Fig. 1:**
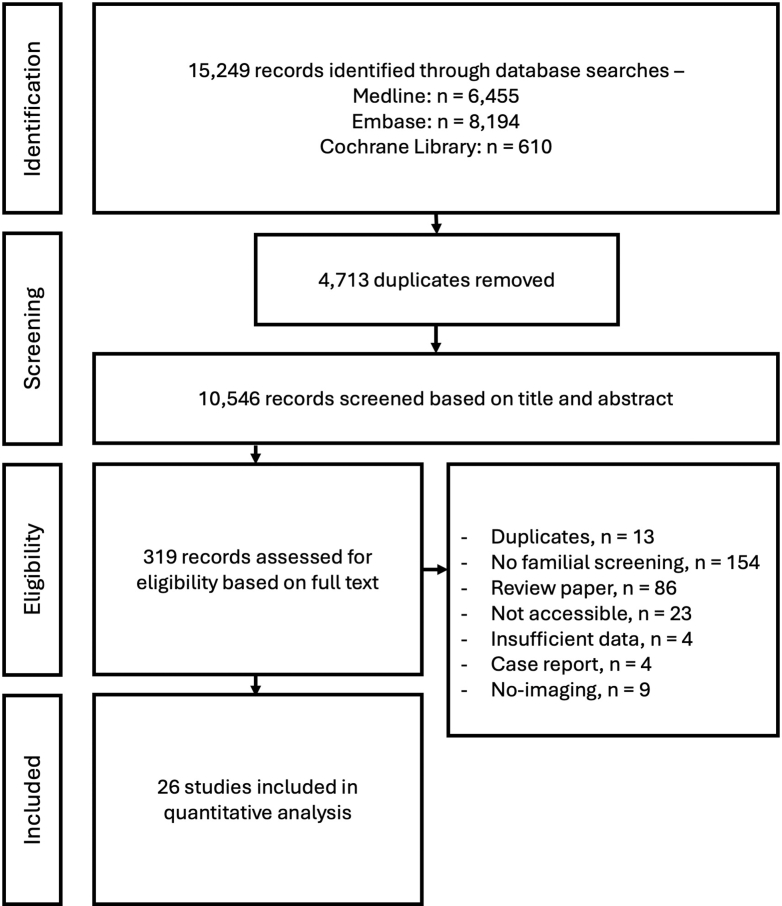
Flow diagram based on the preferred reporting items for systematic reviews and meta-analyses (PRISMA) 2020 guidelines showing the method of identifying trials and reasons for exclusion.

Twenty-six studies met the criteria for inclusion in this meta-analysis (Fig. 1). All were retrospective observational studies^16^, ^17^, ^18^, ^19^, ^20^, ^21^^,^^36^, ^37^, ^38^, ^39^, ^40^, ^41^, ^42^, ^43^, ^44^, ^45^, ^46^, ^47^, ^48^, ^49^, ^50^, ^51^, ^52^, ^53^, ^54^, ^55^ and the majority were full texts (24/26).^16^, ^17^, ^18^, ^19^, ^20^, ^21^^,^^36^^,^^38^, ^39^, ^40^, ^41^, ^42^, ^43^, ^44^, ^45^, ^46^^,^^48^, ^49^, ^50^, ^51^, ^52^, ^53^, ^54^, ^55^ The studies were performed in 11 different countries (Sweden,^21^^,^^36^^,^^40^^,^^52^^,^^54^ Netherlands,^42^^,^^48^^,^^49^^,^^55^ USA,^38^ Canada,^20^^,^^41^^,^^44^ UK,^16^^,^^17^^,^^39^ Australia,^19^^,^^46^ Belgium,^53^ Finland,^18^^,^^47^ Ireland,^45^^,^^51^ Italy,^37^^,^^43^ New Zealand^50^), and published between 1989 and 2024, including 4166 AAA index patients and 4530 screened relatives (Table 1). AAA index patients had an average age of 70.0 ± 9.6 years, and 82.1% (1872/2280) were male. Only one study reported AAA index patient co-morbidities, where 14% (45/322) had diabetes, 50% (161/322) had hypertension, and 39% (126/322) smoked.^53^ The proportion of screened relatives who responded to invitation was 65.7% (3900/5939), with an average age of 63.6 ± 8.2 years (range: 53.2 to 69.4), and 47.6% (1890/3970) were male. Of the studies reporting screened relative co-morbidities, 8.5 ± 4.1% (234/2763) had diabetes,^20^^,^^21^^,^^36^^,^^43^^,^^48^^,^^49^^,^^51^, ^52^, ^53^, ^54^ 32.1 ± 11.1% (1438/4480) had hypertension^20^^,^^21^^,^^36^^,^^43^^,^^45^^,^^48^^,^^49^^,^^51^, ^52^, ^53^, ^54^ and 29.7 ± 20.6% (873/2941) smoked.^20^^,^^21^^,^^36^^,^^39^^,^^43^^,^^45^^,^^48^^,^^49^^,^^51^, ^52^, ^53^, ^54^ Nine studies included FDRs, twelve included only siblings, two included FDRs and second-degree relatives (SDRs), two included only offspring, and one included only brothers (Table 1). Overall, of the 4530 screened relatives, 4451 were FDRs, and 79 were SDRs.

Nine non-imaging studies were excluded, reporting data based on interviews and questionnaires. These were not included in the main analyses, but a sensitivity analysis was performed to allow for comparison between different methodologies.

Of the 26 included studies, all 26 were considered to have a ‘low’ risk of bias (Supplementary Table S4). The leading area of bias was non-response bias, affecting 54% (14/26) of the included studies, of which people living too far from screening centres and direct refusal were significant contributors. Overall, all outcomes were assessed to be of moderate certainty due to heterogeneity potentially causing inconsistency.

**Fig. 2:**
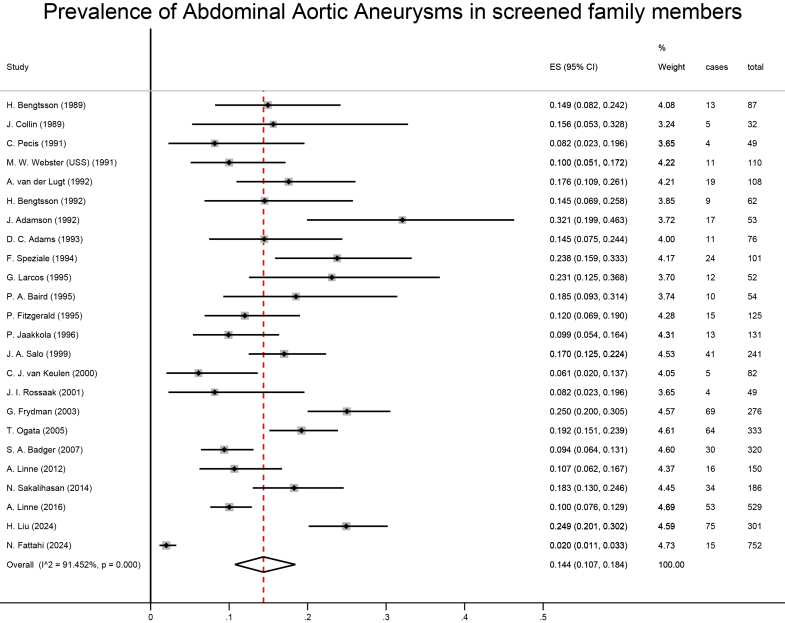
Forest plot showing the pooled prevalence of all screened relatives having an abdominal aortic aneurysm. ES, effect size; CI, confidence interval.

There were 24 included studies which reported the prevalence of AAA in both male and female relatives, with a total number of screened relatives of 4259.^16^, ^17^, ^18^, ^19^, ^20^, ^21^^,^^36^, ^37^, ^38^, ^39^, ^40^^,^^42^, ^43^, ^44^, ^45^, ^46^, ^47^^,^^49^, ^50^, ^51^, ^52^, ^53^, ^54^, ^55^ Of those relatives, 569 were identified as having AAA, with a pooled prevalence of 14.4% (95% CI 10.7%–18.4%, I^2^ 91% p < 0.001) (Fig. 2).

**Fig. 3:**
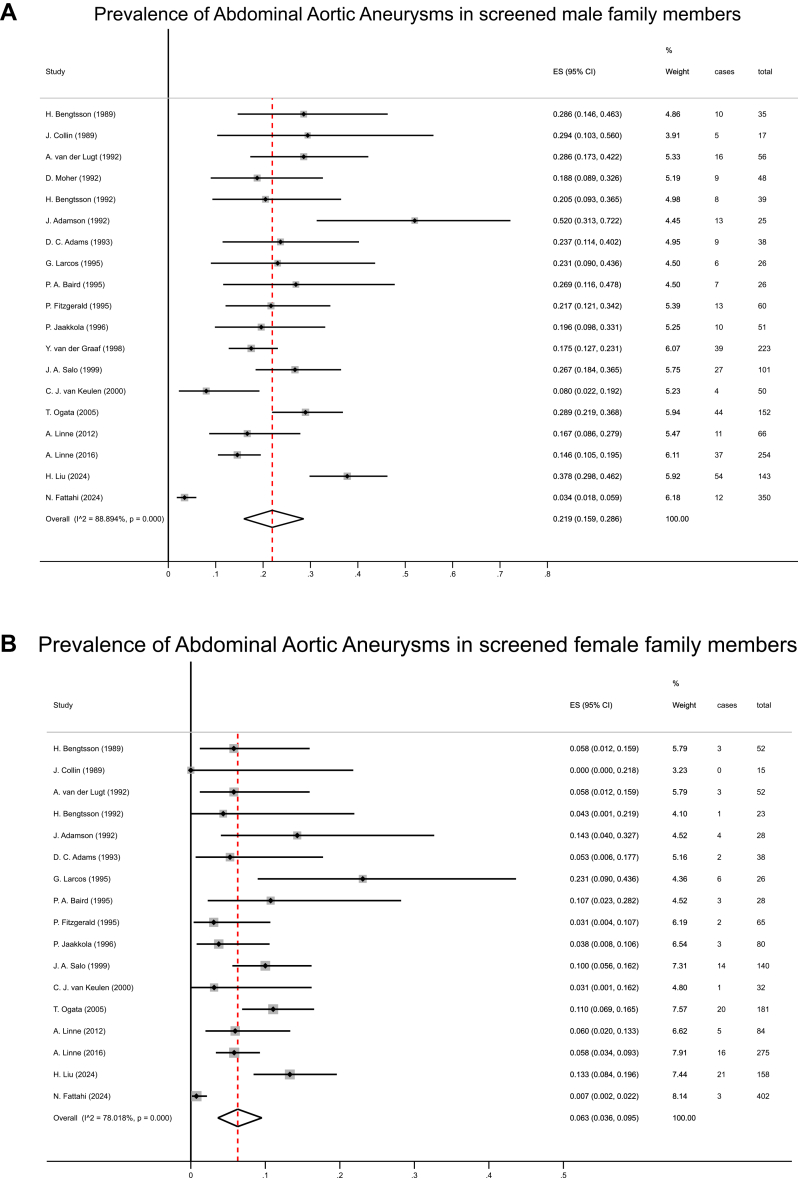
Forest plots showing the pooled prevalence of screened male (**A**) and female (**B**) relatives having an abdominal aortic aneurysm. ES, effect size; CI, confidence interval.

**Fig. 4:**
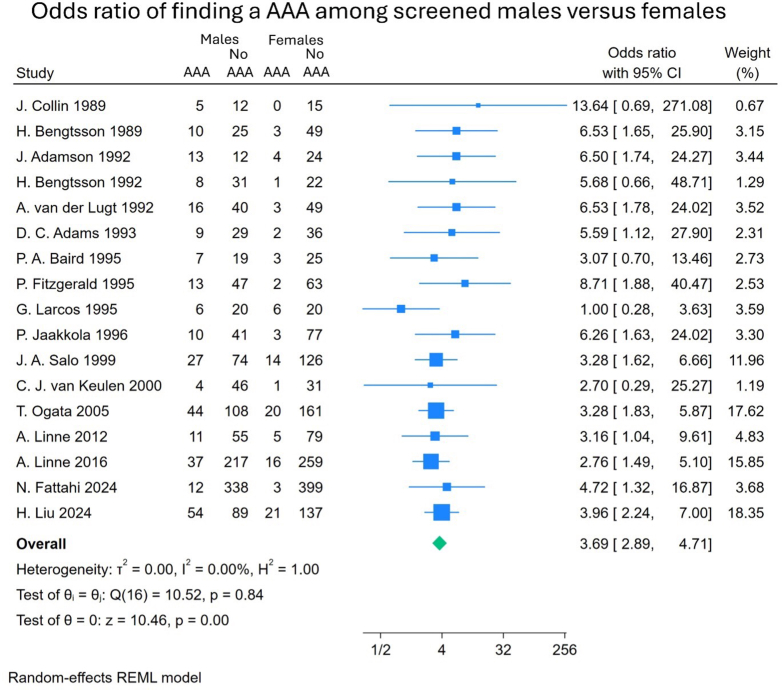
Forest plot showing the odds of finding an abdominal aortic aneurysm amongst screened males versus screened females. AAA, abdominal aortic aneurysm; CI, confidence interval.

Of the 24 studies, 17 reported the sex of the screened relatives,^16^, ^17^, ^18^^,^^20^^,^^21^^,^^36^^,^^39^^,^^40^^,^^42^^,^^44^, ^45^, ^46^, ^47^^,^^49^^,^^52^^,^^54^^,^^55^ and two additional studies only included males.^41^^,^^48^ Of 1760 screened male relatives, 334 were identified as having AAA, with a pooled prevalence of 21.9% (95% CI 15.9%–28.6%, I^2^ 89% p < 0.001) (Fig. 3A). From the 17 studies reporting the sex of the screened relatives, 1679 female relatives were screened, and 107 were identified as having AAA, with a pooled prevalence of 6.3% (95% CI 3.6%–9.5%, I^2^ 78% p < 0.001) (Fig. 3B). In meta-analysis comparing male and female prevalence, screened males had about four times greater odds of having an AAA than females (OR 3.69, 95% CI 2.89–4.71, I^2^ 0% p > 0.05) (Fig. 4).

**Fig. 5:**
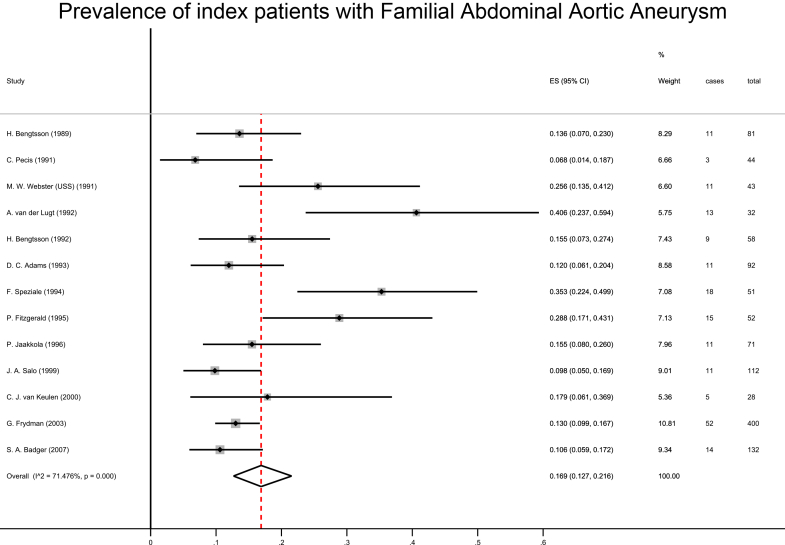
Forest plot showing the pooled prevalence of index patients with familial AAA (number of AAA index patients with at least one affected relative). AAA, abdominal aortic aneurysm; ES, effect size; CI, confidence interval.

Thirteen included studies reported the number of AAA index patients with at least one newly affected screened relative.^17^, ^18^, ^19^^,^^36^, ^37^, ^38^^,^^40^^,^^42^^,^^43^^,^^45^^,^^47^^,^^49^^,^^51^ Of 1196 AAA index patients, 184 were found to be familial, having at least one affected relative. The pooled prevalence was 16.9% (95% CI 12.7%–21.6%, I^2^ 71% p < 0.001) (Fig. 5).

Five studies also reported the AAA prevalence in control groups, such as the screened relatives’ spouses, for both males and females,^18^, ^19^, ^20^^,^^44^^,^^54^ including 1346 controls with a mean age of 65.5 ± 8.0 years, and 47.8% (643/1346) were male. Furthermore, one study reported data for only male controls (Supplementary Table S5).^41^ Of the 1346 controls screened, 21 were identified to have an AAA, giving a pooled prevalence of 2.8% (95% CI 0.6%–6.4%, I^2^ 88% p < 0.001). When including the additional study only screening male controls, of 759 males, 31 were found to have an AAA, giving a pooled prevalence of 4.1% (95% CI 0.7%–9.4%, I^2^ 86% p < 0.001). Of the 703 female controls screened, 4 were found to have an AAA, giving a pooled prevalence of 0.2% (95% CI 0.0%–0.9%, I^2^ 0% p = 0.854) (Supplementary Table S6). The relative risk for males is 3.17 (95% CI 1.39–7.23, five studies) and for females is 5.03 (95% CI 1.88–13.5, five studies).

Seven included studies reported individual data for 16 females found to have AAA. We have summarised the characteristics of these cases in Supplementary Table S7, as there is currently limited evidence to guide policy when screening female relatives.

The 26 included studies did not report enough data on rates of intervention, morbidity or mortality to perform clinical outcome-related analysis. Subgroup analysis was based on AAA size definition and relative definition (Table 2). Sensitivity analyses were performed based on the removal of enriched cohorts such as those including AAA index patients with known familial AAA, analysis methodology and overlapping study populations (Table 2). Overall, these variables did not grossly affect the outcomes. There was no correlation in overall prevalence with the middle of each study's recruitment timepoints, suggesting prevalence amongst screened relatives did not change over time (Supplementary Figure S1). Nor was there an association between AAA index patient sex and relative AAA prevalence (Supplementary Table S8). All included studies were identified as having a low risk of bias. No evidence of small-study effects were seen using funnel plots and Egger's regression for each outcome (Supplementary Figures S2–S5). There was a significant difference in prevalence between countries of origin; however, this subgroup analysis did not reduce overall heterogeneity (Supplementary Figure S6). Sensitivity analyses were also performed examining the four main outcomes based on nine non-imaging familial AAA prevalence studies to allow comparison with imaging versus questionnaire/interview methodology. For the prevalence in all relatives, the outcome was significantly lower than that of the imaging studies. For male relatives and female relatives alone, only 2 studies reported outcomes, and for familial AAA, the prevalence was similar to that of the imaging studies.^12^, ^13^, ^14^, ^15^^,^^57^, ^58^, ^59^, ^60^, ^61^

Multiple meta-regressions of AAA prevalence amongst screened individual relatives, versus relevant reported study-level covariates, did not explain the observed heterogeneity (Supplementary Table S8) (covariates: age, sex, diabetes, hypertension and smoking status).

## Discussion

This meta-analysis is both large and comprehensive, providing moderate-certainty evidence, evaluating the prevalence of AAA in screened family members of patients with AAA. This review encompasses data from 26 studies, with data collected from 4530 relatives. Approximately 1 in 7 of all screened relatives, 1 in 5 screened males, and 1 in 16 screened females were found to have AAA. Almost 1 in 6 AAA index patients were identified as having at least one affected relative (familial AAA). Men included in this study had about four times the odds of being found to have an AAA versus the females. Nevertheless, the females (n = 1679) were at a 5 times greater risk of being screened to have an AAA compared with matched controls (n = 703), and the males (n = 1760) were at approximately 3 times greater risk, compared with matched controls (n = 640).

We compared screened relatives with control groups from the included studies because these provide a more appropriate comparator than external population studies, given differences in geography, recruitment period and screening methodology between cohorts. The control prevalence estimates were also broadly consistent with reported population prevalence data, supporting the generalisability of the included studies.^11^^,^^68^, ^69^, ^70^

The markedly increased prevalence of AAA observed amongst relatives in this meta-analysis supports the importance of genetic susceptibility in AAA pathogenesis. Familial aggregation has consistently been demonstrated in family history,^12^, ^13^, ^14^, ^15^ screening^16^, ^17^, ^18^, ^19^, ^20^, ^21^ and twin studies.^23^^,^^24^ Our finding that almost 1 in 6 AAA index patients had at least one affected relative further reinforces the contribution of inherited risk. Although environmental factors such as smoking remain important contributors to AAA development, the persistently elevated prevalence observed amongst relatives, including females and across multiple decades of study recruitment, suggests that familial predisposition remains a major determinant of AAA risk.

Subgroup analysis showed that when categorising studies by different AAA diameter cutoffs, the outcomes remained similar. Furthermore, when subgrouping by type of relative, siblings generally show a higher prevalence of AAA, likely due to them being a similar age to the AAA index patients. Very few studies look at the prevalence in offspring alone, and more data are needed to help address their risk as robustly as we have done for siblings. Excluding studies with AAA index patients who had previously known familial AAA, as well as overlapping studies, made very little difference in the outcomes. In our sensitivity analysis, we also analysed the outcomes from nine non-imaging studies which used questionnaires and interviews to investigate AAA prevalence in relatives. As expected, the overall prevalence amongst relatives of 7% was less than our estimate from the imaging studies, due to missing undetected/asymptomatic cases. The figures for male and female relatives alone are based on only two studies and, as a result, are not likely to be accurate. Lastly, the main outcome in the non-imaging studies, which is the prevalence of familial AAA, yielded a similar outcome to the imaging studies, supporting our estimate.

The UK national screening programme focuses exclusively on men turning 65, but states that individuals with a family history of AAA or other risk factors can discuss the possibility of screening with their GP.^5^ In the authors’ experience, this is not standardised. Our findings would tend to support the implementation of national family screening policies, designed to detect aneurysms in this highly enriched cohort to enable timely intervention and prevent deaths from ruptured aneurysms.

Our estimates are significantly greater than the screened prevalence of AAA in the general population of men aged 65 and over, as reported in the NHS AAA screening programme (NAAASP). From 2024 to 2025, 277,991 men were screened, and 1907 (0.69%) were found to have AAA.^69^ Based on our male prevalence, compared with the national screening prevalence, the results suggest that male relatives are 32 times more enriched for AAA than the general population of men aged 65 and above, the only population in which national routine screening is currently implemented.^27^, ^28^, ^29^, ^30^ Moreover, screened female relatives are approximately 9 times more enriched for AAA, when comparing our female prevalence with a prevalence of 0.74% found in a large meta-analysis of screened women of the general population in the UK.^70^ Given the marked decline in AAA prevalence in the general male population, both in the UK between 2013 and 2023,^10^ and in Sweden, where prevalence fell to <1% in 2022,^11^ targeted screening of this familial-predisposed, highly enriched population may enhance the effectiveness of current screening programmes. Interestingly, our sensitivity analysis showed that despite reductions in the prevalence of AAA in the general populations of the countries included in our analysis,^10^^,^^71^ the prevalence of familial AAA does not appear to have declined between the 1980s and 2020s. This supports the hypothesis that, on a population level, familial risk of AAA remains a strong risk factor, potentially counteracting the protective effect of reduced environmental risk factors, such as smoking, thereby supporting targeted screening of family members of patients with AAA. More work is needed to identify the ideal age to screen for familial AAA; however, the average age among screened relatives where AAA were found was 63.6 years, and specifically 61.2 years for individually reported female cases, the only age data currently available for females alone.

Current international guideline recommendations advise offering screening to high-risk individuals, including those with a positive family history, regardless of sex.^27^, ^28^, ^29^, ^30^ Our data support this, but given the minimal change in outcomes when excluding studies with AAA index patients who had previously known familial AAA, our data also support screening all relatives of patients with AAA, regardless of a positive family history. Family histories can be inaccurate, families may not share medical information between members, and asymptomatic cases are missed. Therefore, given the significant risk and lack of awareness among relatives,^31^ alongside the declining cost-effectiveness of current national screening programmes, there is an argument to increase the screening of relatives of all patients with AAA, irrespective of them reporting a positive family history. Furthermore, considering the significant risk posed to relatives, further genetic studies would be helpful to increase our understanding of this association.

We found a relatively high proportion of missing study-level data, particularly participant characteristics in the form of risk factors, amongst screened relatives. This hampered our attempts to perform sensitivity analysis through meta-regression. There was insufficient data to perform imputation, in accordance with the Cochrane handbook. Additionally, with incomplete screening responses, there is a possibility of loss of generalisability and a possibility of non-response bias. Some studies may duplicate reporting of the same population, but sensitivity analyses removing sources of overlap resulted in similar outcomes. Screening programmes rely on principles such as the Wilson criteria to ensure a programme achieves a net overall benefit.^72^ When it comes to family screening for AAA, policy may benefit from further data on clinical outcomes among screened relatives found to have AAA. Nonetheless, we can still draw parallels with non-familial screening of AAA.

This meta-analysis provides a comprehensive and reliable estimate of the prevalence of AAA amongst screened relatives, showing that when screened from the age of early 50s to late 60s, they are a highly enriched population for the detection of AAA. Screened male relatives have a high prevalence of AAA, but female relatives are also at risk, and we found the highest relative risks in female relatives, compared to controls of the same sex. Despite screened familial women having a greater prevalence of AAA than men of the general population screened at 65, familial-predisposed women do not currently receive routine, systematic screening. This meta-analysis provides pooled estimates of the prevalence of AAA amongst screened relatives, to guide targeted screening strategies and support clinical counselling for both male and female relatives, irrespective of reported family history in the AAA index patient.

## Contributors

LB acted as guarantor and was involved in study design, screening, data collection, data analysis, draughting of the manuscript, reviewing and editing of the final manuscript. JJHB was involved in supervision of the project, study design and conceptualisation, search strategy, data analysis, reviewing and editing of the final manuscript. CEF-A was involved in the risk of bias assessment, reviewing and commenting on draughts of the final manuscript. SET was involved in screening, reviewing and commenting on draughts of the final manuscript. DH was involved in study design and conceptualisation, reviewing and commenting on draughts of the final manuscript. AP was involved in study design and conceptualisation, reviewing and commenting on manuscript draughts, including the final manuscript. All authors had full access to the data in the study and accept responsibility for the decision to submit for publication. Both LB and JJHB accessed and verified all underlying data.

## Data sharing statement

Data is available upon reasonable request from the corresponding author.

## Declaration of interests

AP received honoraria and support for travel from GE Healthcare. All other authors have none to declare.
