# Supplementary material for: Family screening for abdominal aortic aneurysms: a systematic review and meta-analysis
Source: eClinicalMedicine. 2026 Jul 9;97:104056. doi: 10.1016/j.eclinm.2026.104056 (PMC13380107; doi:10.1016/j.eclinm.2026.104056)
Supplement: Supplement [file mmc1.docx]

**Supplementary materials**

# Table of Contents

[Table of Contents 1](#_Toc232451385)

[Supplementary Table 2 – PICO model to determine research question 2](#_Toc232451386)

[Supplementary Table 3 – abdominal aortic aneurysm AND familial screening search strategy 2](#_Toc232451387)

[Supplementary Table 4 – Risk of bias assessment tool for prevalence studies (Hoy *et al*, 2012) 6](#_Toc232451388)

[Supplementary Table 5 – Control data 9](#_Toc232451389)

[Supplementary Table 6 – Control outcomes 9](#_Toc232451390)

[Supplementary Table 7 – Individual female data 10](#_Toc232451391)

[Supplementary Figure 1 – Meta-regression assessing the middle of each study’s recruitment time point to assess the change in prevalence of AAA in relatives over time. 11](#_Toc232451392)

[Supplementary Figure 2 – Funnel plot assessing small study bias for the prevalence of abdominal aortic aneurysms in screened family members (24 studies) 12](#_Toc232451393)

[Supplementary Figure 3 – Funnel plot assessing small study bias for the prevalence of abdominal aortic aneurysms in screened male family members (19 studies) 12](#_Toc232451394)

[Supplementary Figure 4 – Funnel plot assessing small study bias for the prevalence of abdominal aortic aneurysms in screened female family members (17 studies) 13](#_Toc232451395)

[Supplementary Figure 5 – Funnel plot assessing small study bias for the prevalence of index patients with familial abdominal aortic aneurysm (13 studies) 13](#_Toc232451396)

[Supplementary Figure 6 – Forest plot showing the overall prevalence of screened relatives found to have an AAA subgrouped by study country of origin 14](#_Toc232451397)

[Supplementary Table 8 – Meta-regressions 15](#_Toc232451398)

# Supplementary Table 2 – PICO model to determine research question

| **PICO Tool** | **Description** |
| --- | --- |
| **Population** | Relatives of patients with abdominal aortic aneurysms |
| **Intervention** | The use of screening using imaging |
| **Comparison** | The general population or non-relatives |
| **Outcome** | The pooled prevalence of abdominal aortic aneurysms in screened relatives of patients with abdominal aortic aneurysms. |

**Supplementary table 2**: Outline of the model used to formulate the research question.

# Supplementary Table 3 – abdominal aortic aneurysm AND familial screening search strategy

| **EMBASE Search** | | |
| --- | --- | --- |
| Embase <1974 to 4 March 2026> | | |
| **#** | **Query** | **Results** |
| 1 | exp abdominal aortic aneurysm/ | 41,109 |
| 2 | abdominal aortic aneurysm.ti,ab. | 24,536 |
| 3 | AAA.ti,ab. | 26,996 |
| 4 | 1 or 2 or 3 | 55,460 |
| 5 | exp mass screening/ | 383,085 |
| 6 | exp early diagnosis/ | 154,226 |
| 7 | exp genetic disorder/ | 1,752,290 |
| 8 | exp quantitative trait/ | 10,767 |
| 9 | exp nuclear family/ | 407,875 |
| 10 | screen*.ti,ab. | 1,741,076 |
| 11 | famil*.ti,ab. | 1,906,922 |
| 12 | relative*.ti,ab. | 2,358,315 |
| 13 | (heritable or heritability).ti,ab. | 58,893 |
| 14 | inherited.ti,ab. | 155,351 |
| 15 | 5 or 6 or 7 or 8 or 9 or 10 or 11 or 12 or 13 or 14 | 7,487,572 |
| 16 | 4 and 15 | 9,998 |
| 17 | limit 16 to human | 8,194 |
| **MEDLINE Search** | | |
| Ovid MEDLINE(R) ALL <1946 to 4 March 2026> | | |
| **#** | **Query** | **Results** |
| 1 | exp Aortic Aneurysm, Abdominal/ge [Genetics] | 1,094 |
| 2 | exp Aortic Aneurysm, Abdominal/ | 24,687 |
| 3 | abdominal aortic aneurysm.ti,ab. | 18,689 |
| 4 | AAA.ti,ab. | 18,651 |
| 5 | 2 or 3 or 4 | 38,330 |
| 6 | exp Mass Screening/ | 155,087 |
| 7 | exp Early Diagnosis/ | 80,589 |
| 8 | exp Genetic Diseases, Inborn/ | 777,240 |
| 9 | exp Quantitative Trait, Heritable/ | 9,422 |
| 10 | exp Nuclear Family/ | 6,113 |
| 11 | screen*.ti,ab. | 1,196,732 |
| 12 | famil*.ti,ab. | 1,467,160 |
| 13 | relative*.ti,ab. | 1,885,228 |
| 14 | (heritable or heritability).ti,ab. | 46,123 |
| 15 | inherited.ti,ab. | 108,300 |
| 16 | 6 or 7 or 8 or 9 or 10 or 11 or 12 or 13 or 14 or 15 | 5,047,817 |
| 17 | 5 and 16 | 6,126 |
| 18 | 1 or 17 | 6,927 |
| 19 | 18 not (Animals/ not (Animals/ and Humans/)) | 6,455 |
| **COCHRANE Search** | | |
| Date Run: 4 March 2026 | | |
| **#** | **Query** | **Results** |
| 1 | MeSH descriptor: [Aortic Aneurysm, Abdominal] explode all trees | 897 |
| 2 | Abdominal Aortic Aneurysm | 1,691 |
| 3 | AAA | 2,271 |
| 4 | #1 OR #2 OR #3 | 3,287 |
| 5 | MeSH descriptor: [Mass Screening] explode all trees | 6,251 |
| 6 | MeSH descriptor: [Early Diagnosis] explode all trees | 3,677 |
| 7 | screen* | 127,307 |
| 8 | MeSH descriptor: [Genetic Diseases, Inborn] explode all trees | 18,843 |
| 9 | MeSH descriptor: [Quantitative Trait, Heritable] explode all trees | 51 |
| 10 | famil* | 83,313 |
| 11 | (heritable or heritability) | 558 |
| 12 | first-degree relative* | 1,329 |
| 13 | FDR | 1,202 |
| 14 | first degree relative* | 6,419 |
| 15 | inherited | 2,441 |
| 16 | #5 OR #6 OR #7 OR #8 OR #9 OR #10 OR #11 OR #12 OR #13 OR #14 OR #15 | 219,406 |
| 17 | #4 AND #16 | 610 |

# Supplementary Table 4 – Risk of bias assessment tool for prevalence studies (Hoy *et al*, 2012)

|  |  | **Risk of bias criteria** | | | | | | | | | | |
| --- | --- | --- | --- | --- | --- | --- | --- | --- | --- | --- | --- | --- |
|  |  | **External validity** | | | | **Internal validity** | | | | | | |
| **First author** | **Year** | **1** | **2** | **3** | **4** | **5** | **6** | **7** | **8** | **9** | **10** | **Total** |
| J. Collin | 1989 | 1 | 1 | - | 0 | 1 | 0 | 1 | 1 | 1 | 1 | 7 |
| H. Bengtsson | 1989 | 1 | 1 | 1 | 1 | 1 | 1 | 1 | 1 | 1 | 1 | 10 |
| C. Pecis | 1991 | - | 1 | 1 | - | 1 | - | 1 | 1 | 1 | 1 | 7 |
| M. W. Webster (USS) | 1991 | 1 | 1 | 0 | 0 | 1 | 1 | 1 | 1 | 1 | 1 | 8 |
| J. Adamson | 1992 | - | 1 | 1 | 1 | 1 | 1 | 1 | 1 | 1 | 1 | 9 |
| H. Bengtsson | 1992 | 1 | 1 | 1 | 0 | 1 | 1 | 1 | 1 | 1 | 1 | 9 |
| D. Moher | 1992 | 1 | 1 | - | 1 | 1 | 1 | 1 | 1 | 1 | 1 | 9 |
| A. van der Lugt | 1992 | 1 | 1 | - | 0 | 1 | 1 | 1 | 1 | - | 1 | 7 |
| D. C. Adams | 1993 | 1 | 1 | 1 | 0 | 1 | 1 | 1 | 1 | 1 | 1 | 9 |
| F. Speziale | 1994 | 1 | 1 | - | 0 | 1 | 1 | 1 | 1 | 1 | 1 | 8 |
| P. A. Baird | 1995 | 1 | 1 | 1 | 1 | 1 | 1 | 0 | 0 | 1 | 1 | 8 |
| P. Fitzgerald | 1995 | 1 | 1 | 1 | 0 | 1 | 1 | 1 | 1 | 1 | 1 | 9 |
| G. Larcos | 1995 | - | 1 | 1 | 0 | 1 | 1 | 1 | 1 | 1 | 1 | 8 |
| P. Jaakkola | 1996 | 1 | 1 | 1 | 0 | 1 | - | 1 | 0 | 1 | 1 | 7 |
| Y. van der Graaf | 1998 | 1 | 1 | - | 1 | 1 | 1 | 1 | 1 | 1 | 1 | 9 |
| J. A. Salo | 1999 | 1 | 1 | 1 | 1 | 1 | 1 | 1 | 1 | 1 | 1 | 10 |
| C. J. van Keulen | 2000 | 1 | 1 | 1 | 1 | 1 | 1 | 1 | 1 | 1 | 1 | 10 |
| J. I. Rossaak | 2001 | 1 | 1 | 1 | 1 | 1 | 1 | 1 | 1 | 1 | 1 | 10 |
| G. Frydman | 2003 | 1 | 1 | 1 | 0 | 1 | 1 | 1 | 1 | 1 | 1 | 9 |
| T. Ogata | 2005 | 1 | 1 | 1 | 0 | 1 | 1 | 1 | 1 | 1 | 1 | 9 |
| S. A. Badger | 2007 | 1 | 1 | 1 | 1 | 1 | 1 | 1 | 1 | 1 | 1 | 10 |
| A. Linne | 2012 | 1 | 1 | 1 | 1 | 1 | 1 | 1 | 1 | 1 | 1 | 10 |
| N. Sakalihasan | 2014 | 1 | 1 | - | 0 | 1 | 1 | 1 | 1 | 1 | 1 | 8 |
| A. Linne | 2016 | - | 1 | 1 | 1 | 1 | 1 | 1 | 1 | 1 | 1 | 9 |
| N. Fattahi | 2024 | 1 | 1 | 1 | 1 | 1 | 1 | 1 | 1 | 1 | 1 | 10 |
| H. Liu | 2024 | 1 | 1 | 1 | 0 | 1 | 1 | 1 | 1 | 1 | 1 | 9 |
| **Total** | | **22** | **26** | **19** | **12** | **26** | **23** | **25** | **24** | **25** | **26** | **228** |

**Supplementary table 4**: Table showing the risk of bias scores using the Hoy *et al* tool for prevalence studies. Studies received a score of 1 for each question they satisfied, or 0 if they did not. “-“ denotes insufficient information. An overall score of 7-10, 4-6, and 0-3 represents ‘low’, ‘moderate’, and ‘high’ risk of bias, respectively. Numbered items 1-4 refer to external validity and 5-10 to internal validity:

- - - 1. Was the study’s target population a close representation of the national population in relation to relevant variables?
      2. Was the sampling frame a true or close representation of the target population?
      3. Was some form of random selection used to select the sample, OR was a census undertaken?
      4. Was the likelihood of nonresponse bias minimal?
      5. Were data collected directly from the subjects (as opposed to a proxy)?
      6. Was an acceptable case definition used in the study?
      7. Was the study instrument that measured the parameter of interest shown to have validity and reliability?
      8. Was the same mode of data collection used for all subjects?
      9. Was the length of the shortest prevalence period for the parameter of interest appropriate?
      10. Were the numerator(s) and denominator(s) for the parameter of interest appropriate?

# Supplementary Table 5 – Control data

| **Study** | **Type of control** | **Number of controls screened (male/female)** | **Number with AAA (male/female)** |
| --- | --- | --- | --- |
| D. Moher 1992 | Cataract patients | 116 (male only) | 14 (14/-) |
| P. A. Baird 1995 | Cataract patients | 100 (56/44) | 8 (8/0) |
| J. A. Salo 1999 | Unclear but patients matched for sex and age with no AAA family history | 284 (135/149) | 4 (2/2) |
| G. Frydman 2003 | Pateints  having abdominal CT  scans for non-vascular indications | 118 (61/57) | 0 (0/0) |
| T. Ogata 2005 | Siblings of index patient spouses | 88 (42/46) | 2 (2/0) |
| N. Fattahi 2024 | Identified from national registry | 756 (349/407) | 7 (5/2) |

**Supplementary table 5:** Table showing all included studies which reported control data, and the data collected for meta-analysis. AAA, abdominal aortic aneurysm; CT, computed tomography.

# Supplementary Table 6 – Control outcomes

| **Control outcome** | **Prevalence % (95% CI)** | **I2 %, p** |
| --- | --- | --- |
| Prevalence of abdominal aortic aneurysms in screened controls, n = 5^(36, 41, 43, 57, 66)^ | 2.8 (0.6-6.4) | 87.99, p < 0.001 |
| Prevalence of abdominal aortic aneurysms in screened male controls, n = 6^(36, 41, 43, 51, 57, 66)^ | 4.1 (0.7-9.4) | 85.62, p < 0.001 |
| Prevalence of abdominal aortic aneurysms in screened female controls, n = 5^(36, 41, 43, 57, 66)^ | 0.2 (0.0-0.9) | 0.00, p = 0.854 |

**Supplementary table 6:** Table showing pooled prevalence for the three outcomes analysed from the control data. “n” denotes the number of studies. CI: confidence interval.

# Supplementary Table 7 – Individual female data

| **Author** | **Year** | **Identifier** | **Relation to proband** | **Age (years)** | **AAA size (cm)** | **Repair?** | **Rupture?** |
| --- | --- | --- | --- | --- | --- | --- | --- |
| H. Bengtsson | 1989 | A | Sister | 61 | 2.8 | No | No |
|  |  | B | Sister | 71 | 3.6 | No | No |
|  |  | C | Sister | 74 | 3.3 | No | No |
| M. W. Webster | 1991 (USS study) | A | Sister | 62 | 2.8 | No | No |
| H. Bengtsson | 1992 | A | Daughter | 68 | 3.1 | No | No |
| F. Speziale | 1994 | A | Sister | 67 | 2.7 | No | No |
|  |  | B | Sister | 57 | 2.7 | No | No |
| P. Fitzgerald | 1995 | A | Sister | 58 | Not reported but study definition >3cm | Not reported | No |
|  |  | B | Sister | 78 | Not reported but study definition >3cm | Not reported | No |
| G. Larcos | 1995 | A | Daughter | 55 | 2.8 | No | No |
|  |  | B | Sister | 52 | 2.6 | No | No |
|  |  | C | Daughter | 41 | 2.5 | No | No |
|  |  | D | Sister | 63 | 2.5 | No | No |
|  |  | E | Daughter | 50 | 2.7 | No | No |
|  |  | F | Daughter | 54 | 2.6 | No | No |
| C. J. van Keulen | 2000 | A | Sister | 68 | Not reported | Yes | Not clear |

**Supplementary table 7**: Table showing the individual female data reported in the included studies where AAA size ≥ 2.5cm.

# Supplementary Figure 1 – Meta-regression assessing the middle of each study’s recruitment time point to assess the change in prevalence of AAA in relatives over time.

**
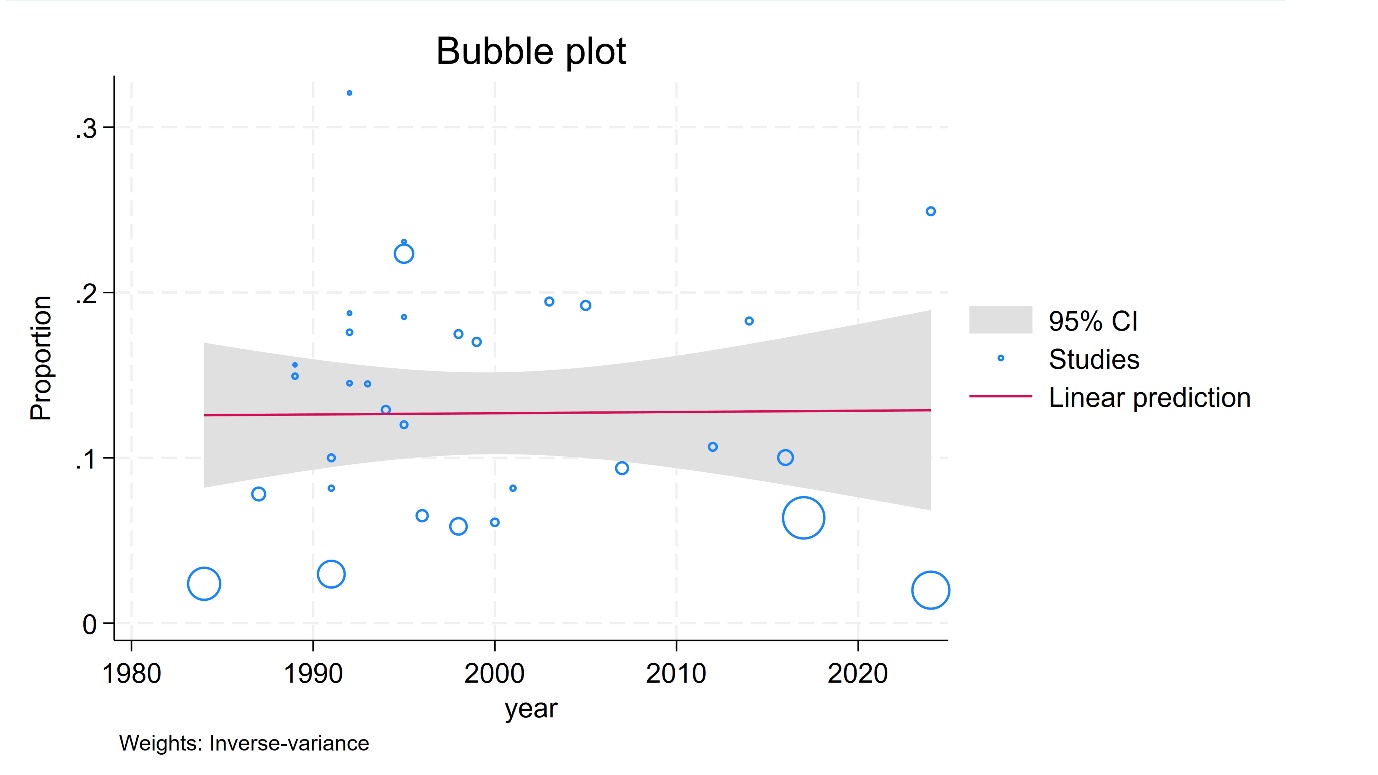
**

# Supplementary Figure 2 – Funnel plot assessing small study bias for the prevalence of abdominal aortic aneurysms in screened family members (24 studies)

**
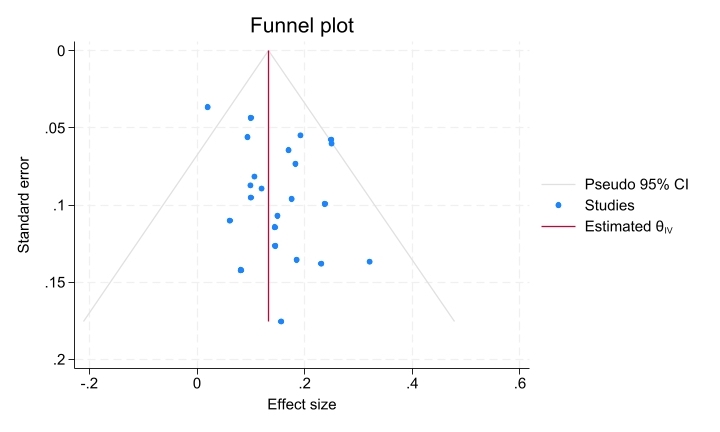
**

# Supplementary Figure 3 – Funnel plot assessing small study bias for the prevalence of abdominal aortic aneurysms in screened male family members (19 studies)

**
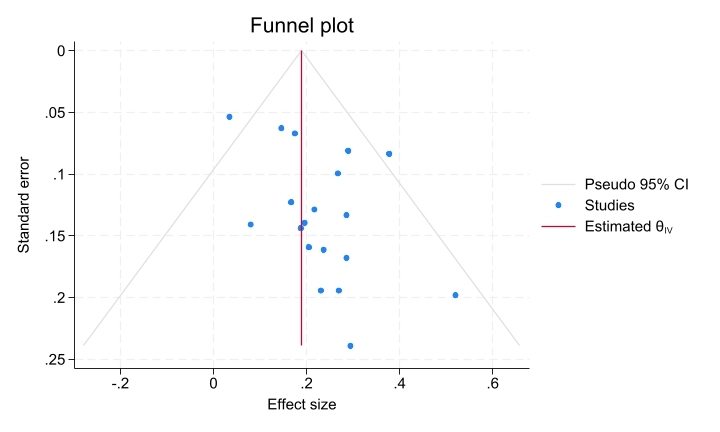
**

# Supplementary Figure 4 – Funnel plot assessing small study bias for the prevalence of abdominal aortic aneurysms in screened female family members (17 studies)

**
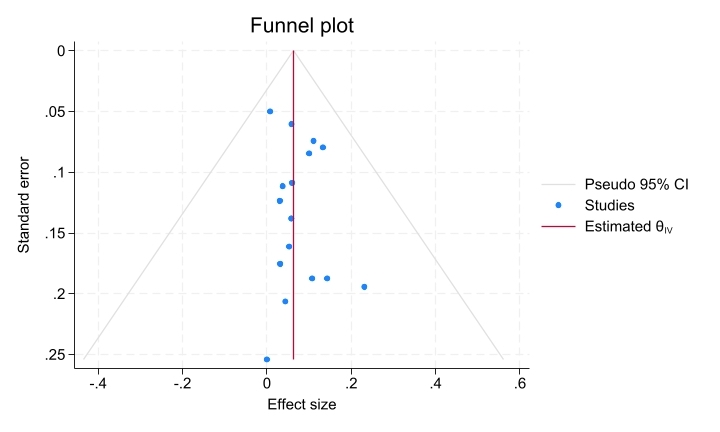
**

# Supplementary Figure 5 – Funnel plot assessing small study bias for the prevalence of index patients with familial abdominal aortic aneurysm (13 studies)


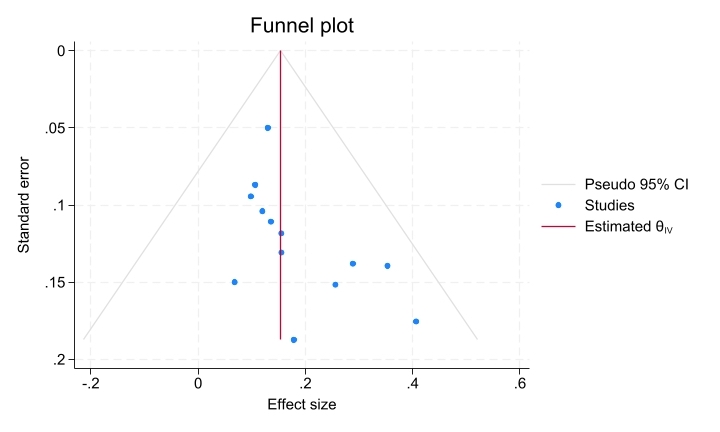


# Supplementary Figure 6 – Forest plot showing the overall prevalence of screened relatives found to have an AAA subgrouped by study country of origin


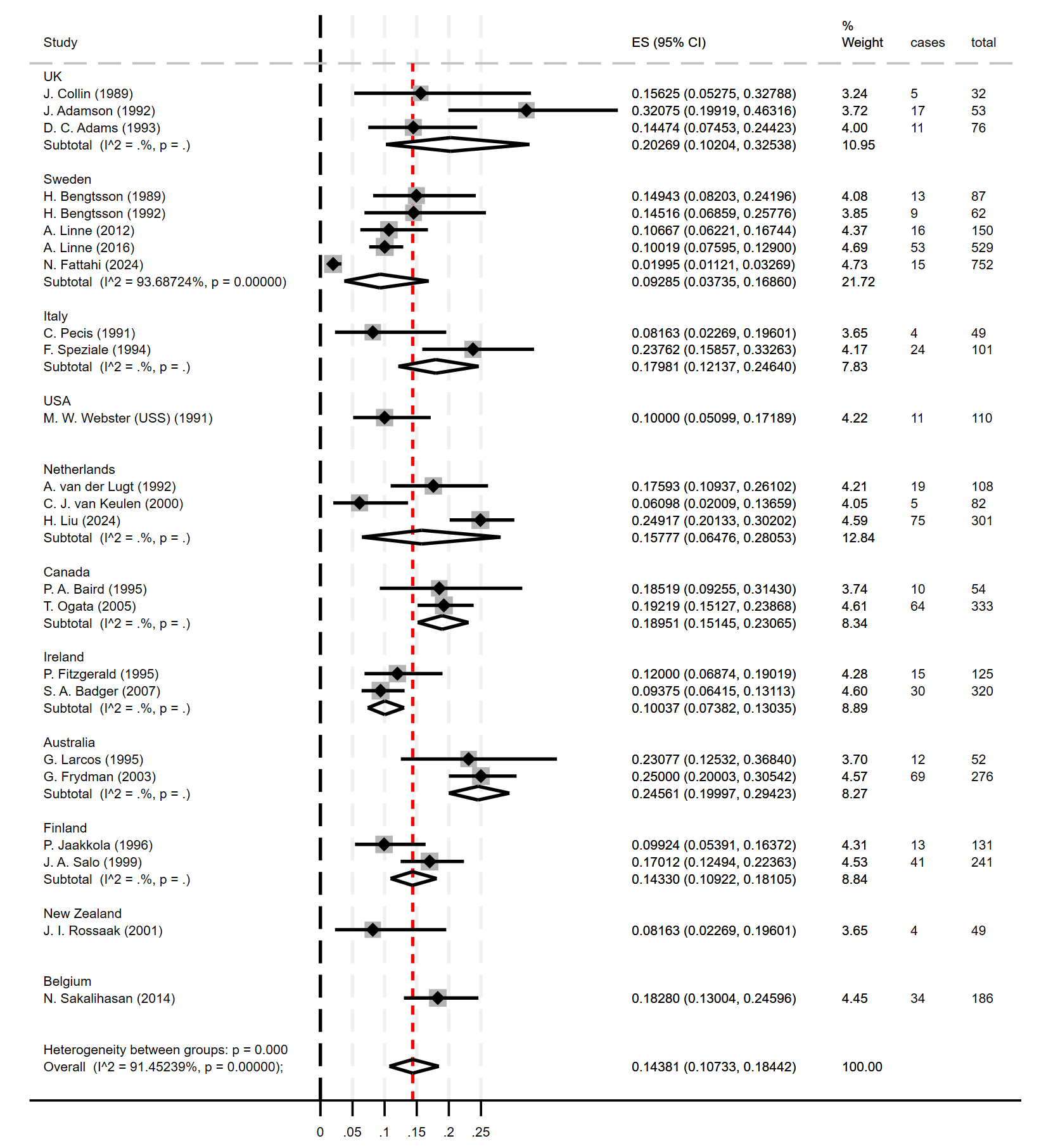


# Supplementary Table 8 – Meta-regressions

| **Covariate** | **N** | **Coefficient (95% CI)** | **P value** |
| --- | --- | --- | --- |
| **Univariate** | | | |
| Age (years) | 17 | 0.001 (-0.007 to 0.01) | 0.75 |
| Sex (% male) | 20 | -0.0004 (-0.001 to 0.003) | 0.89 |
| Index sex (% male) | 12 | 0.003 (-0.0009 to 0.008) | 0.12 |
| Diabetes (%) | 9 | 0.002 (-0.01 to 0.013) | 0.78 |
| Hypertension (%) | 10 | -0.001 (-0.006 to 0.003) | 0.52 |
| Smoker (%) | 11 | 0.002 (-0.0003 to 0.004) | 0.10 |
| **Multivariate** | | | |
| Age (years) | - | -0.003 (-0.03 to 0.03) | 0.84 |
| Sex (% male) | - | -0.007 (-0.02 to 0.008) | 0.35 |
| Diabetes (%) | - | 0.009 (-0.02 to 0.04) | 0.57 |
| Hypertension (%) | - | -0.006 ( -0.01 to 0.003) | 0.19 |
| Smoker (%) | - | 0.002 (-0.0004 to 0.004) | 0.12 |
| Constant | 8 | 0.72 (-1.75 to 3.18) | 0.57 |

**Supplementary table 8:** Univariate meta-regressions on different prevalences of screened abdominal aortic aneurysms against study-level covariates. CI, confidence interval. *Index sex omitted from multivariate meta-regression due to collinearity.
